# Automated PROMISE V2 Scoring from PSMA PET/CT Reports Using Large Language Models: A Comparative Evaluation of Prompt Design and Model Performance

**DOI:** 10.3390/curroncol33060349

**Published:** 2026-06-09

**Authors:** Tilman Speicher, Isa Ethem Demirkol, Arne Blickle, Moritz B. Bastian, Stephan Maus, Andrea Schaefer-Schuler, Mark Bartholomä, Caroline Burgard, Samer Ezziddin, Florian Rosar

**Affiliations:** 1 Department of Nuclear Medicine, Saarland University-Medical Center, 66421 Homburg, Germany; 2 Department of Nuclear Medicine, Friedrich-Alexander-Universität Erlangen-Nürnberg and Universitätsklinikum Erlangen, 91054 Erlangen, Germany

**Keywords:** PROMISE, LLM, large language model, prostate cancer, PSMA, PET/CT

## Abstract

Large language models (LLMs) are increasingly explored for clinical use. However, the extent to which such models can reliably support physicians in reporting, staging, and the assessment of classification remains an active area of research. This study aimed to evaluate and compare multiple LLMs for automated PROMISE V2 classification for prostate cancer. A total of 126 unambiguous German-language PSMA PET/CT text reports were retrospectively analyzed, with reference standards established by expert consensus based on image interpretation and the original report text. Five LLMs (GPT-5.4, DeepSeek-V3.2, Claude Sonnet 4.6, Gemini 3 Flash and Grok 4) were assessed using two English-language prompting strategies of varying complexity. Agreement with the reference standard served as the primary endpoint. Performance varied in the short-prompt setting (36.5–79.4%) but improved consistently with the long prompt (74.6–86.5%), with Gemini 3 Flash achieving the highest agreement. Across PROMISE V2 subcategories, agreement rates were high (miT: 81.0–92.1%, miN: 92.9–96.0%, miM: 92.9–95.2%), despite inter-model differences. In conclusion, contemporary LLMs demonstrate promising performance in deriving PROMISE V2 scores from unambiguous original report texts, particularly when guided by detailed prompts.

## Simple Summary

This study evaluated whether large language models (LLMs) can accurately derive an established staging score for prostate cancer from free-text PET/CT imaging reports. Five LLMs were tested on 126 unambiguous German-language reports using two English-language prompting strategies. Performance improved consistently with more detailed prompts, with heterogenous agreement rates up to 86.5%. However, the results also indicate notable differences in performance across models and highlight the influence of prompt design on extraction accuracy.

## 1. Introduction

Prostate cancer is among the most prevalent malignancies in men worldwide and is associated with a significant mortality rate [1]. The prostate-specific membrane antigen (PSMA) molecule has emerged as a widely utilized molecular target in both diagnostic and therapeutic modalities, establishing it as a central component of contemporary theranostic strategies [2,3,4,5,6,7,8]. For staging and treatment monitoring in prostate cancer, PSMA-targeted positron emission tomography/computed tomography (PET/CT) currently represents the most important imaging modality. This method enables the detection of even small lesions and, through precise anatomical localization, facilitates targeted therapeutic approaches and improves patient outcomes [9,10], although reporting of individual cases in free-text format can be complex because of marked interpatient variability in tumor burden, extent of involvement, and metastatic sites. To standardize staging, the second version of the ‘*Prostate Cancer Molecular Imaging Standardized Evaluation Framework Including Response Evaluation for Clinical Trials*’ (PROMISE V2) is widely applied [11]. The framework proposes criteria for whole-body staging designed to characterize the extent of prostate cancer based on PSMA PET/CT imaging. These criteria encompass PSMA-targeted molecular imaging-derived local tumor burden (T), regional nodal involvement (N), and the presence of distant metastases (M), collectively forming an imaging-based analog of the well-established TNM staging system. The PROMISE V2 catalog provides clinicians and clinical researchers with a robust framework to characterize disease status in a standardized and reproducible manner. In parallel, clinicians stand to benefit from streamlined, digitally optimized workflows, particularly when accompanied by the integration of AI-supported processes that enhance efficiency and consistency. Molecular imaging classifications are becoming increasingly important, also beyond clinical routine, e.g., in multicenter research collaborations and theranostic concepts, when data needs to be collected in a standardized manner. In multicenter settings in particular, standardized scoring systems such as PROMISE V2 improve interinstitutional comparability and enable more reliable comparison of imaging-derived staging information across different centers and study populations. However, despite the existence of structured and well-defined classification frameworks such as PROMISE V2, a relevant fraction of clinical reports continues to be generated in non-standardized free-text form, which complicates longitudinal assessment and automated data extraction and requires time-consuming manual interpretation by treating physicians or researchers. With regard to the PROMISE V2 score, the primary clinical benefit of the AI-supported process would lie in the automated extraction of structured scoring information from non-standardized free-text reports. Such an approach could improve clinical workflow efficiency and facilitate more rapid patient assessment by the treating physician. The rapidly increasing availability of large language models (LLMs) developed by multiple commercial providers has enabled broad and convenient global access to advanced artificial intelligence technologies. Owing to their strong natural language processing capabilities, LLMs are increasingly being explored for potential applications in medicine, including clinical documentation, structured reporting, automated information extraction, clinical decision support, and workflow optimization [12,13,14,15]. In radiology and nuclear medicine in particular, the ability of LLMs to process complex free-text reports and transform them into structured classifications has attracted growing scientific interest. Such approaches may contribute to improved reporting standardization, facilitate large-scale retrospective data analysis, and potentially reduce the workload associated with manual extraction of clinically relevant information from imaging report texts. Compared with traditional rule-based natural language processing approaches, contemporary LLMs offer the advantage of flexible contextual interpretation without requiring manually predefined extraction rules for every possible report formulation. Classical keyword- or regex-based extraction systems frequently struggle with variable sentence structure, implicit negation, or complex anatomical descriptions commonly encountered in oncologic imaging report texts [16]. In contrast, LLMs are theoretically capable of integrating contextual information across multiple report sections and handling heterogeneous linguistic formulations. At the same time, the implementation of LLMs in clinical practice remains associated with substantial challenges. Despite their impressive linguistic performance, current models may produce inconsistent outputs or misinterpret context-dependent medical terminology. These limitations are particularly relevant in oncologic imaging report texts, where subtle differences in wording may directly affect disease staging, therapeutic stratification, and treatment planning. Furthermore, the performance of LLMs may vary considerably depending on prompt design, model architecture, and task complexity, highlighting the need for systematic validation in clinically relevant scenarios [17]. Despite the rapidly growing number of studies investigating LLM applications in medicine, only limited evidence is currently available regarding their use in imaging classification systems such as PROMISE V2. Previously published studies have focused on text report summarization, rather than comprehensive text-based staging frameworks [18]. Consequently, it remains uncertain to what extent current LLMs can reliably reproduce expert-level PROMISE V2 staging from free-text PSMA PET/CT reports under clinically realistic conditions. Consequently, this study aims to evaluate, compare, and validate multiple LLMs (GPT-5.4, Gemini 3 Flash, Grok 4, DeepSeek-V3.2, Claude Sonnet 4.6) for the automated assessment of PROMISE V2, based on free-text PSMA PET/CT reports.

## 2. Methods

This analysis investigates the ability of different LLMs to determine the PROMISE V2 score in patients with prostate cancer based on clinical input using a prompt-engineering approach. As input, the original German-language report text of a PSMA-targeted PET/CT and the corresponding impression text were used without further modification (the score itself has been removed from the text). All inputs were harmonized prior to analysis to ensure consistency across model runs, and any potentially identifying patient information was removed to comply with data protection and ethical standards. All patients agreed to the publication of any data in anonymized form according to the declaration of Helsinki. The analysis was authorized by the Institutional Review Board (Ärztekammer des Saarlandes/Saarbrücken; ethics committee permission number 170/22).

Exclusion criteria comprised reports with uncertain or ambiguous lesion interpretation, low or equivocal tracer uptake, unclear locoregional tumor extension, or indeterminate osseous findings without a corresponding morphologic correlate. In total, 178 PSMA PET/CT reports were retrospectively reviewed, of which *n* = 126 were ultimately included in the study. The remaining 52 examinations were excluded due to ambiguous findings or unclear reporting. All included examinations originated from a university center specialized in prostate cancer imaging and theranostics (Department of Nuclear Medicine, Saarland University—Medical Center, Homburg, Germany). All PSMA PET/CT scans were performed as part of routine clinical care for staging. Among the included cases, 63 PSMA PET/CT scans were performed using [^68^Ga]Ga-PSMA-11 and 63 using [^18^F]DCFPyL. All reference standard evaluations were performed using the complete PSMA PET/CT dataset, including PET, low-dose CT, and the clinical report text. The reviewing physicians had experience in molecular imaging–based prostate cancer staging and applied the PROMISE V2 framework according to current consensus recommendations. Particular attention was paid to anatomically complex disease patterns, equivocal nodal findings, and differentiation between intrapelvic and extrapelvic metastatic involvement. To ensure consistency of the reference standard, all examinations were reviewed jointly in a consensus setting rather than independently, thereby minimizing interobserver variability and establishing a unified final classification for subsequent comparison with LLM-derived outputs. The PROMISE V2 comprises the components miT, miN, and miM for standardized molecular imaging–based staging of prostate cancer. Within the PROMISE V2 framework, miT describes the local tumor extent, miN characterizes regional pelvic nodal involvement, and miM reflects distant metastatic spread. The miM category is subdivided into distant lymph node metastases (miM1a), osseous metastases (miM1b), and visceral metastases (miM1c). In cases of metastatic spread involving multiple anatomical compartments, multiple miM categories were assigned concurrently. Table 1 summarizes the cohort characteristics, including PROMISE V2-based scoring derived from PSMA PET/CT.

The following LLMs were evaluated: GPT-5.4 (Open AI, San Francisco, CA, USA), DeepSeek-V3.2 (Deepseek, Hangzhou, China), Claude Sonnet 4.6 (Anthropic, San Francisco, CA, USA), Gemini 3 Flash (Google Alphabet, San Francisco, CA, USA) and Grok 4 (xAI, Palo Alto, Santa Clara, CA, USA). The evaluated models differed with respect to provider architecture, accessibility, and deployment strategy. GPT-5.4 and Claude Sonnet 4.6 are proprietary closed-source models with a strong emphasis on reasoning performance and enterprise-oriented API deployment, whereas Gemini 3 Flash is integrated within the broader Google AI ecosystem with optimization for scalable multimodal processing and high-throughput inference. Grok 4 is embedded within the xAI platform and emphasizes real-time integration and rapid conversational inference, while DeepSeek-V3.2 represents a comparatively more openly accessible architecture with broader availability for research-oriented applications. The models were available through commercial subscription-based infrastructures. All evaluated LLMs represent contemporary general-purpose multimodal language models optimized for advanced reasoning and natural language processing tasks.

Prior to the submission into the LLM, all reports were converted into a standardized plain-text format. Personally identifiable information, including patient names, dates of birth, examination identifiers, and institutional metadata, was removed using the integrated anonymization function of the Sectra (PACS/RIS software, Sectra workstation IDS7 Version 26.2) reporting environment, followed by manual verification. The English-language prompt was executed between January and March 2026. All models were accessed via their respective application programming interfaces (APIs), and all experimental evaluations were conducted using a Python-based (version 3.11) workflow implemented within the Anaconda environment (version 2.6.3) and developed in the Spyder application (version 6.1.0). A custom script automated the submission of prompts, retrieval of model responses, structured storage of outputs and comparison with the reference standard. The workflow included JSON output formatting and structured storage of model predictions for downstream statistical analysis. No automatic translation of the German report texts was performed prior to inference.

To assess the impact of prompt design on model performance, two different prompt formats were developed with varying levels of detail in the task instruction. The short prompt contained a minimal description of the PROMISE V2 scoring system and consisted of about 250 words, whereas the long prompt included a comprehensive and detailed explanation of the PROMISE V2 scoring system and consisted of about 2000 words. Details of prompt characteristics are summarized in Table 2. Inference parameters characteristics for all LLMs are presented in the supplementary material (Table S1). Each prompt explicitly instructed the LLM to predict the corresponding PROMISE V2 score and to return the result in a standardized JSON format limited to the score value. The long prompt was developed iteratively using a rule-based prompt engineering strategy aimed at minimizing ambiguity in the PROMISE V2 assignment. Particular emphasis during prompt development was placed on hierarchical decision logic and explicit prioritization of PROMISE V2 categories. The long prompt incorporated predefined classification rules for local tumor extension, nodal disease distribution, and distant metastatic involvement, following PROMISE V2, including explicit instructions regarding inconsistent terminology and conflict resolution between findings and impression sections. The short prompt intentionally omitted most of these detailed instructions in order to evaluate the influence of prompt complexity on model performance. Both the complete long and short prompts are provided in the supplementary material.

The prompt types were submitted independently to each model to ensure comparability. Model outputs were recorded without modification. For quality control, all generated outputs were checked for compliance with the predefined JSON structure prior to statistical evaluation, and repeated inference was performed in cases of invalid or incomplete formatting. Outputs were considered valid if they contained all required PROMISE V2 components and used only predefined category labels. The JSON structure consisted exclusively of the three PROMISE V2 categories (‘miT’, ‘miN’, and ‘miM’) and their corresponding classification values in a standardized machine-readable format. This step was performed to ensure that subsequent comparisons reflected classification performance rather than formatting errors. No semantic correction of model responses was performed, and the extracted classifications were compared directly with the expert reference standard.

The primary endpoint was the agreement between the AI-generated PROMISE V2 score and the reference standard. Secondary endpoints focused on evaluating performance differences across prompt formats and LLMs. Descriptive statistics were used to summarize performance metrics (Graph Pad Prism V11, San Diego, CA, USA). Agreement analyses were performed both for the complete composed PROMISE V2 classification and separately for the individual molecular imaging components miT, miN, and miM. Exact agreement was defined as complete concordance between the LLM-derived prediction and the expert-defined reference standard. Cases differing in at least one PROMISE V2 component were considered discordant. Performance comparisons between short and long prompt conditions were conducted descriptively to evaluate the influence of prompt engineering on classification accuracy. Systematic prediction biases were evaluated by analyzing under- and overestimation, followed by the generation of a bias heatmap, defined as the net difference between the two, comparing LLM-derived PROMISE V2 scores against the reference standard. All procedures were conducted in a reproducible computational environment with fixed API parameters, prompt templates, and processing steps.

## 3. Results

**Figure 1:**
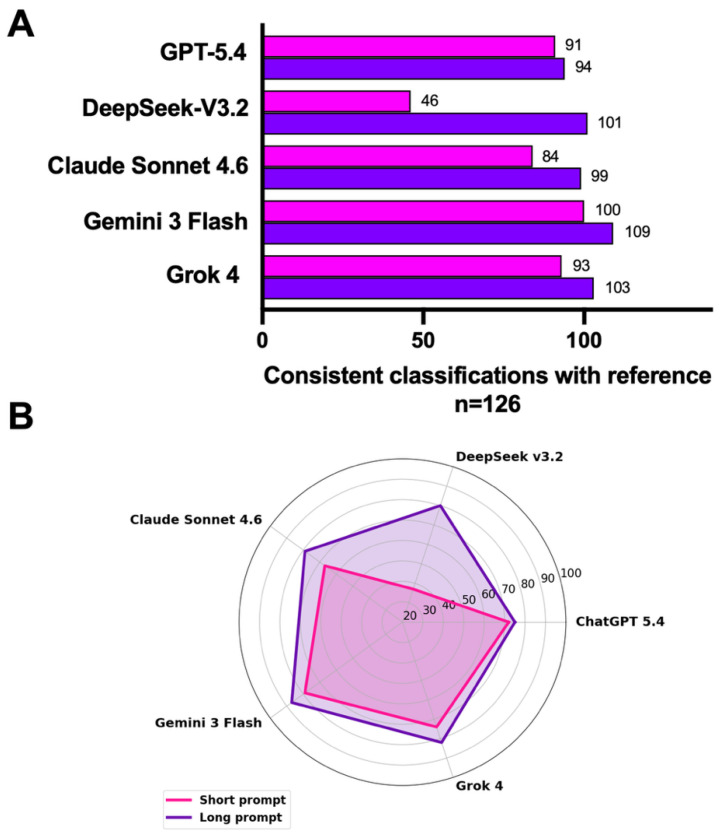
Number (**A**) and proportion (**B**) of consistent classifications with the reference standard across evaluated LLMs.

All evaluated LLMs successfully generated structured outputs for PROMISE V2 score prediction across both prompt formats without technical failures. Agreement of AI-generated PROMISE V2 scores, generated by the initial ‘short prompt’ with the reference standard varied across models with 91/126 correct predictions for GPT-5.4 (72.2%), 46/126 for DeepSeek-V3.2 (36.5%), 84/126 for Claude Sonnet 4.6 (66.7%), 100/126 for Gemini 3 Flash (79.4%), and 93/126 for Grok 4 (73.8%). Attempting to further improve this outcome, a more comprehensive and detailed ‘long prompt’ was developed and implemented. The use of the long prompt resulted in improved agreement with the reference standard compared to the short prompt. Performance improved across all models, with GPT-5.4 archiving agreement with the reference standard in 94/126 (74.6%) cases, representing an improvement of 3 cases compared to the short prompt setting. DeepSeek-V3.2 achieved agreement in 101/126 (80.2%) predictions, representing an increase of 55 consensual classifications; Claude Sonnet 4.6 achieved 99/126 (78.6%), an increase of 15 consensual classifications; Gemini 3 Flash achieved 109/126 (86.5%), an increase of 9 consensual classifications; and Grok 4 achieved 103/126 (81.7%), an increase of 10 consensual classifications. This effect was consistently observed across most PROMISE V2 categories. While the use of the extended prompt resulted in improved performance across all LLMs, the magnitude of this improvement was most pronounced for DeepSeek (Figure 1).

The distribution of consistent classifications for each model and prompt type is illustrated in Figure 1, demonstrating a higher proportion of accurate predictions with the long prompt. The following analyses were continued using the long prompt exclusively.

**Figure 2:**
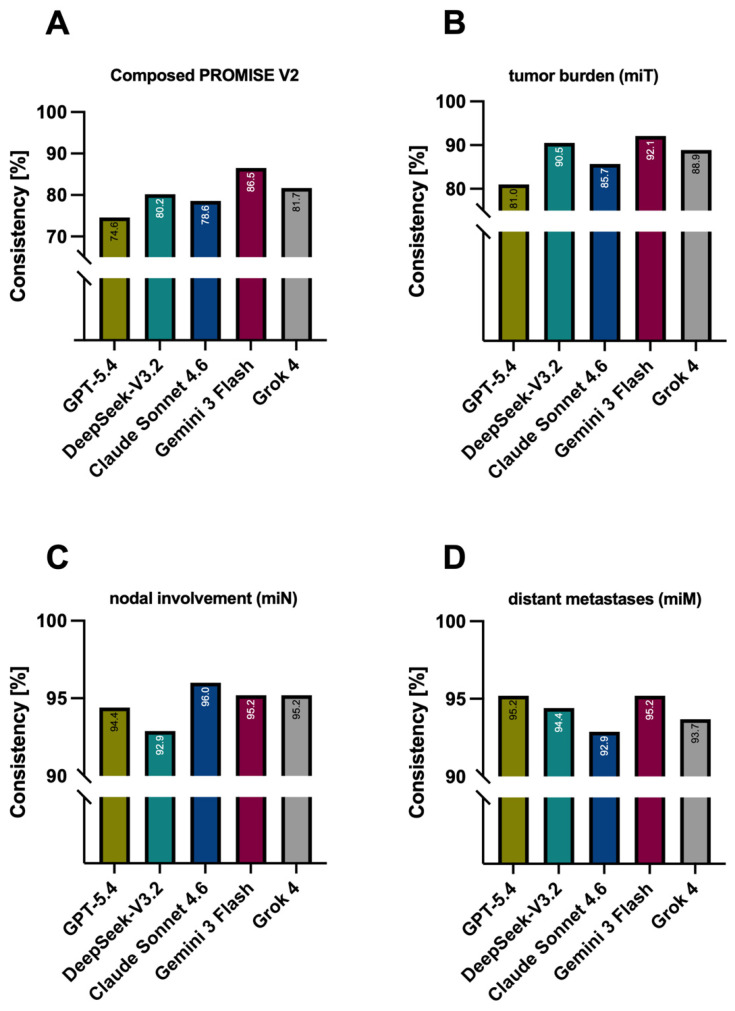
Consistency of LLM generated results for the composed PROMISE V2 score (**A**), and each molecular imaging items: local tumor burden (miT) (**B**), nodal involvement (miN) (**C**) and distant metastases (miM) (**D**) across all evaluated LLMs.

In addition to overall performance, differences were observed across the individual PROMISE V2 categories, miT, miN, and miM classifications, with agreement rates ranging from 81.0% to 92.1% for miT, 92.9% to 96.0% for miN, and 92.9% to 95.2% for miM. These data are presented in detail in Figure 2, which presents the variability in performance across models.

**Figure 3:**
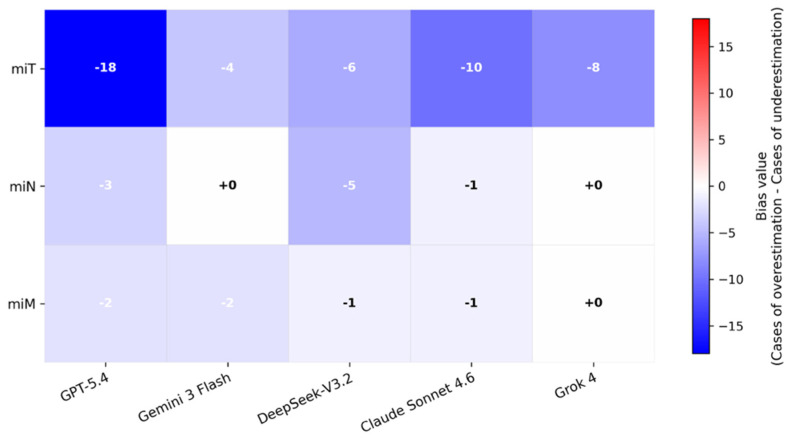
Bias heatmap across evaluated LLMs, calculated by net difference (bias value) of over- and underestimations.

Among inconsistent classifications, all LLMs exhibited both underestimation and overestimation. Table 3 provides a detailed overview of over- and underestimation patterns for each LLM, stratified by miT, miN, and miM ratings. Notably, both DeepSeek-V3.2 and GPT-5.4 seem to exhibit a tendency toward underestimation of the N category. All LLMs tended to underestimate the miT category. This tendency was particularly pronounced for GPT-5.4 and Claude Sonnet 4.6, which underestimated the T category in 21 and 14 cases, respectively. The corresponding heatmap (Figure 3) shows variation in bias values (calculated as net difference between cases of over- and underestimation) across models and categories: miT exhibited consistently negative values across all models (ranging from −18 to −4), which were substantially more negative than those observed for the other categories and indicate a systematic tendency toward underestimation. For miN, several models exhibit negative values, with the lowest value observed for DeepSeek-V3.2 (−5); and for miM, values are closer to zero, with the lowest value at −2 and minor deviations across models. Confusion matrices for all five evaluated LLMs, stratified by miT, miN, and miM categories, are presented in the Supplementary Materials (Figures S1–S15).

## 4. Discussion

In this study, the evaluated LLMs demonstrated a variable capability to derive PROMISE V2 scores from PSMA PET/CT reports, with Gemini 3 Flash presenting the highest accuracy, particularly when using a detailed prompt. A key finding is the substantial improvement in performance across all models when transitioning from a short to a long prompt, highlighting the critical importance of prompt design for complex clinical classification tasks. This suggests that LLMs rely heavily on clear and structured instructions to accurately interpret medical report data. Using the extended prompt, Gemini 3 Flash achieved the highest accuracy in agreement with the reference standard (109/126 cases), while agreement rates ranged from 74.6% to 86.5% across models, despite the use of identical input data and prompting strategies, potentially reflecting differences in underlying model architectures. In addition, the magnitude of improvement achieved through prompt refinement varied considerably across models, with DeepSeek-V3.2 showing the largest gain. These findings may reflect differences in model architecture, training data composition, and instruction-following capabilities.

Overall, the results reported in the literature are largely in agreement with the findings of the present study. Recent studies support the findings and suggest that LLMs are particularly promising for structured oncologic classification tasks based on free-text imaging reports. In a closely related study on neuroendocrine tumors, Mergen et al. showed that LLMs such as ChatGPT-4o, DeepSeek-V3, Claude Sonnet 3.5, and Gemini 2.0 Flash were able to derive UICC and ENETS TNM staging from PET/CT reports with high performance, particularly for N and M classification, while T classification remained more challenging [19]. Similar results were reported for breast cancer staging from PET/CT reports, where Spitzl et al. found high overall performance across several LLMs, with Claude Sonnet 3.5 achieving particularly strong results for T, N, M, and overall UICC stage classification [20]. In prostate cancer specifically, Ismayilov et al. demonstrated that LLMs can accurately stage PSMA PET/CT reports when guided by expert-informed prompt engineering, achieving accuracies above 90% for composite staging tasks [21]. Our results are closely aligned with those of Suzuki et al., who assessed GPT-4 for TNM classification of pancreatic cancer using Japanese radiology reports. Their study likewise demonstrated high performance in N and M classification, whereas accuracy for T classification was comparatively lower, indicating that local tumor assessment may represent a recurring difficulty across different tumor entities [22]. Furthermore, in a recent study, Voltin evaluated the performance of ChatGPT-4 in predicting lymphoma staging based on 80 PET/CT reports, reporting an overall accuracy of 59.0%, indicating a certain variability in accuracy when different tumor entities are considered [23].

Another aspect examined in this study concerns the systematic over- and underestimation exhibited by the different LLMs, which was quantified using a bias metric. The observed bias patterns indicate that LLM performance varies across PROMISE V2 components. For the miT category, a general pronounced tendency toward underestimation was observed across models; while GPT-5.4 and Claude Sonnet 4.6 showed especially high deviations. The local tumor assessment within the miT category may represent a particularly demanding task for LLM-based extraction because these classifications often depend on subtle distinctions regarding extracapsular extension, seminal vesicle invasion, or infiltration of adjacent anatomical structures. Such features are frequently described using nuanced descriptive terminology rather than explicit statements. Consequently, accurate PROMISE V2 assignment requires not only identification of relevant keywords but also interpretation of hierarchical anatomical relationships and contextual clinical wording. Similarly, the predominantly negative values observed for miN suggest a systematic tendency toward underestimation, particularly for DeepSeek-V3.2. For miM, values remain mostly close to zero, indicating largely balanced predictions with limited directional bias toward underestimation. However, these findings should be interpreted as preliminary and hypothesis-generating, and more comprehensive error analyses will be required to delineate a robust hierarchy of model performance. Nonetheless, the unidirectional error pattern is a relevant aspect of the results, with LLMs tending to underestimate rather than overestimate disease extent. One possible explanation is that, when confronted with ambiguity or insufficiently explicit wording, LLMs may adopt a more conservative classification strategy and favor lower-stage categories unless stronger evidence for advanced disease is present in the report text.

From a clinical perspective, automated PROMISE V2 scoring could support standardized reporting and reduce workload in the future. However, even for the best-performing LLM, Gemini 3 Flash, the residual error rate of 13.5% remains clinically relevant, particularly in oncologic decision-making. This concern is amplified in cases of disagreement-related stage shifts, where reclassification may directly alter therapeutic decision-making and treatment strategy. Therefore, LLM outputs should currently be considered assistive rather than definitive. This distinction is particularly important because PROMISE V2 classification may influence clinically relevant communication between nuclear medicine physicians, urologists, radiation oncologists, and medical oncologists. Even when LLM-generated classifications are largely accurate, final validation by a physician remains essential before incorporation into clinical decision-making. Therefore, the most realistic near-term application is not autonomous staging, but supervised workflow support.

Nevertheless, even partially automated extraction of structured staging information may offer substantial practical benefits in high-volume clinical environments. Potential applications include automated pre-population of structured reports, support of multidisciplinary tumor board preparation, facilitation of retrospective registry studies, and acceleration of clinical trial screening processes [24]. In addition, automated extraction of PROMISE V2 classifications from historical free-text reports could substantially simplify the generation of structured research databases for outcome analysis and therapy evaluation.

Interestingly, the overall agreement rates observed in our study (up to 86.5%) were comparable to those reported by Dennstädt et al. (89.2% for Regex and 87.7% for LLMs [25]), despite notable differences in both the clinical domain and reporting framework under investigation. While Dennstädt et al. evaluated the extraction of BI-RADS categories in breast cancer imaging and directly compared rule-based algorithms with LLM-based approaches, our study focused on LLM-based extraction of PROMISE scores from PSMA PET/CT reports. Taken together, these findings suggest that both rule-based and LLM-based methods can achieve similarly high performance when extracting information from highly standardized reporting systems. Nevertheless, given their greater flexibility and scalability, LLM-based approaches are likely to play an increasingly important role in future clinical applications of natural language processing.

Summarizing, the integration of LLMs into clinical workflows for automated prediction of the PROMISE score from report texts holds considerable promise for enhancing efficiency and standardization, but also entails risks related to misclassification, variability in reporting, and the necessity for continued clinical oversight.

Several limitations of this analysis should be acknowledged. The retrospective design and reliance on single-center report texts may limit generalizability. Additionally, model performance is dependent on prompt design and reflects only the specific model versions tested. An important limitation of this study is the cross-lingual nature of the evaluation (German-language input text vs. English-language prompts). This cross-linguistic setup may have influenced model performance, as subtle nuances, domain-specific terminology, or contextual meanings could be lost or misinterpreted during implicit translation processes within the models. Although modern LLMs are generally capable of handling multilingual input, their performance may still vary depending on language combinations and training data distribution. Of note, the observed concordance may have also been biased by class imbalance, as 68.3% of patients were classified as miN0 or miM0 in the reference standard. This likely favored agreement, as identifying the presence or absence of metastatic disease is less demanding than accurate localization and categorization of involved sites. Additionally, due to the joint evaluation of PET/CT images by the three nuclear medicine specialists, the possibility of consensus bias cannot be excluded. Future studies should evaluate whether fully language-consistent setups or explicitly controlled translation pipelines lead to improved accuracy and more robust results, while another potential area for future research could be the evaluation of structured vs. unstructured reporting formats and their impact on model performance. Another important area for future investigation involves the integration of multimodal AI systems capable of directly incorporating imaging information in addition to report text. While the present study exclusively evaluated text-based PROMISE V2 extraction, future multimodal approaches may allow simultaneous analysis of PET/CT images and corresponding narrative reports, potentially improving robustness in ambiguous cases. Such developments may further expand the role of AI-assisted staging within nuclear medicine and oncologic imaging workflows.

## 5. Conclusions

Contemporary large language models (LLMs) show promising but heterogeneous performance in deriving PROMISE V2 scores from unambiguous PSMA PET/CT report texts. Although model-specific differences exist, prompt design emerges as a key factor influencing accuracy, with detailed prompts substantially improving performance.

## Figures and Tables

**Table 1:** Cohort characteristics.

Characteristic	*N* (%)
Number of patients	126 (100%)
[^68^Ga]Ga-PSMA-11	63 (50.0%)
[^18^F]DCFPyL	63 (50.0%)
PROMISE V2 Score	
miT0	10 (7.9%)
miT2u	22 (17.4%)
miT2m	44 (34.9%)
miT3a	3 (2.4%)
miT3b	23 (18.3%)
miT4	19 (15.1%)
miTr	5 (4.0%)
miN0	86 (68.3%)
miN1	9 (7.1%)
miN2	31 (24.6%)
miM0	86 (68.3%)
miM1a	15 (11.9%)
miM1b	18 (14.3%)
miM1c	2 (1.6%)
miM1a-miM1b	4 (3.2%)
miM1a-miM1c	1 (0.8%)

**Table 2:** Prompt characteristics.

Category	Short Prompt	Long Prompt
Role definition	No explicit role	Persona pattern
Complexity	Concise	Detailed, rule-based
Output	JSON	JSON
Rule structure	Key points	Hierarchical decision rules + priorities
Linguistic nuances	Not considered	Explicit rules for medical phrasing
Weighting of the assessment	None	Assessment weighted more heavily than findings
Prioritization	None	Explicit order (miT4 > miT3 > …)
miT-classification	Very coarse	Complete decision logic
miM-classification	Simple	Multiple categories, Combination logic, Strict rules
Anatomical precision	Broad classification	High-precision differentiation

**Table 3:** Over- and underestimations across evaluated LLMs for all patients.

Rating	Category	GPT-5.4	Gemini 3 Flash	Deepseek V3.2	Claude Sonnet 4.6	Grok 4
	miT	21 (16.7%)	7 (5.6%)	9 (7.1%)	14 (11.1%)	11 (8.7%)
Underestimation	miN	5 (4.0%)	3 (2.4%)	7 (5.6%)	3 (2.4%)	3 (2.4%)
	miM	4 (3.2%)	4 (3.2%)	4 (3.2%)	5 (4.0%)	4 (3.2%)
	miT	3 (2.4%)	3 (2.4%)	3 (2.4%)	4 (3.2%)	3 (2.4%)
Overestimation	miN	2 (1.6%)	3 (2.4%)	2 (1.6%)	2 (1.6%)	3 (2.4%)
	miM	2 (1.6%)	2 (1.6%)	3 (2.4%)	4 (3.2%)	4 (3.2%)

## Data Availability

The data supporting the findings of this study are not publicly available but can be obtained from the corresponding author upon reasonable request.

## Supplementary Materials

The following supporting information can be downloaded at: https://www.mdpi.com/article/10.3390/curroncol33060349/s1, Table S1: Inference parameters characteristics for LLM; Figure S1: Confusion Matrix of GPT-5.4 for PROMISE Score V2 Classification (miM); Figure S2: Confusion Matrix of GPT-5.4 for PROMISE Score V2 Classification (miN); Figure S3: Confusion Matrix of GPT-5.4 for PROMISE Score V2 Classification (miT); Figure S4: Confusion Matrix of Claude Sonnet 4.6 for PROMISE Score V2 Classification (miM); Figure S5: Confusion Matrix of Claude Sonnet 4.6 for PROMISE Score V2 Classification (miN); Figure S6: Confusion Matrix of Claude Sonnet 4.6 for PROMISE Score V2 Classification (miT); Figure S7: Confusion Matrix of DeepSeek V3.2 for PROMISE Score V2 Classification (miM); Figure S8: Confusion Matrix of DeepSeek V3.2 for PROMISE Score V2 Classification (miN); Figure S9: Confusion Matrix of DeepSeek V3.2 for PROMISE Score V2 Classification (miT); Figure S10: Confusion Matrix of Gemini 3 Flash for PROMISE Score V2 Classification (miM); Figure S11: Confusion Matrix of Gemini 3 Flash for PROMISE Score V2 Classification (miN); Figure S12: Confusion Matrix of Gemini 3 Flash for PROMISE Score V2 Classification (miT); Figure S13: Confusion Matrix of Grok 4 for PROMISE Score V2 Classification (miM); Figure S14: Confusion Matrix of Grok 4 for PROMISE Score V2 Classification (miN); Figure S15: Confusion Matrix of Grok 4 for PROMISE Score V2 Classification (miT).
