# Supplementary material for: Automated PROMISE V2 Scoring from PSMA PET/CT Reports Using Large Language Models: A Comparative Evaluation of Prompt Design and Model Performance
Source: Curr Oncol. 2026 Jun 9;33(6):349. doi: 10.3390/curroncol33060349 (PMC13298828; doi:10.3390/curroncol33060349)
Supplement: Supplementary file 1 [file curroncol-33-00349-s001.zip › curroncol-4254804-supplementary.pdf]

Table S1. Inference parameters characteristics for LLM.

Model	Temperature	Top_p	Max tokens	Seed
OpenAI GPT-5.4	1.0	1.0	no fixed default	random by default
DeepSeek-V3.2	1.0	1.0	no fixed default	random by default
Gemini 3 Flash	1.0	1.0	no fixed default	random by default
Claude Sonnet 4.6	1.0	1.0	no fixed default	random by default
Grok-4	1.0	1.0	no fixed default	random by default

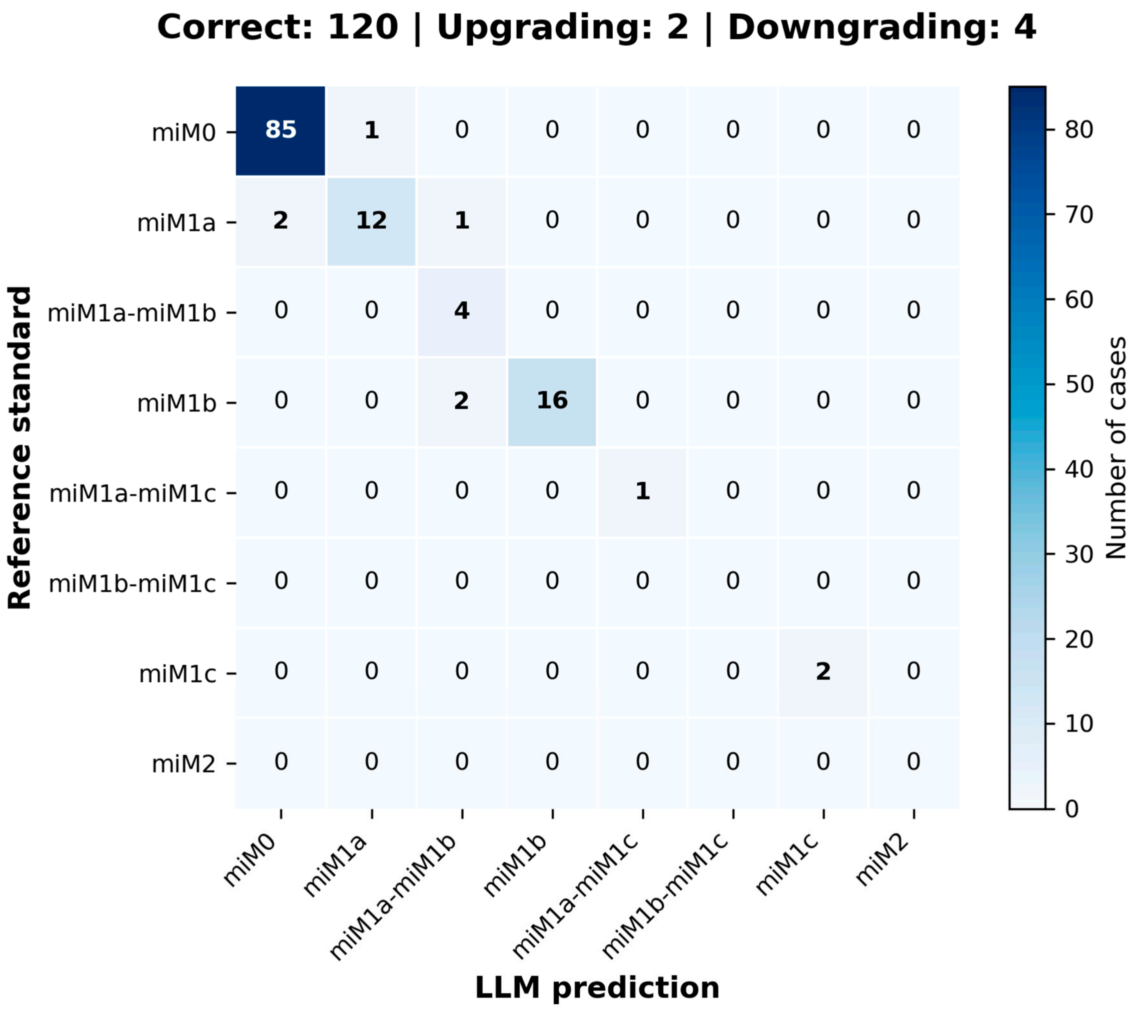

Figure S1. Confusion Matrix of GPT-5.4 for PROMISE Score V2 Classification (miM).

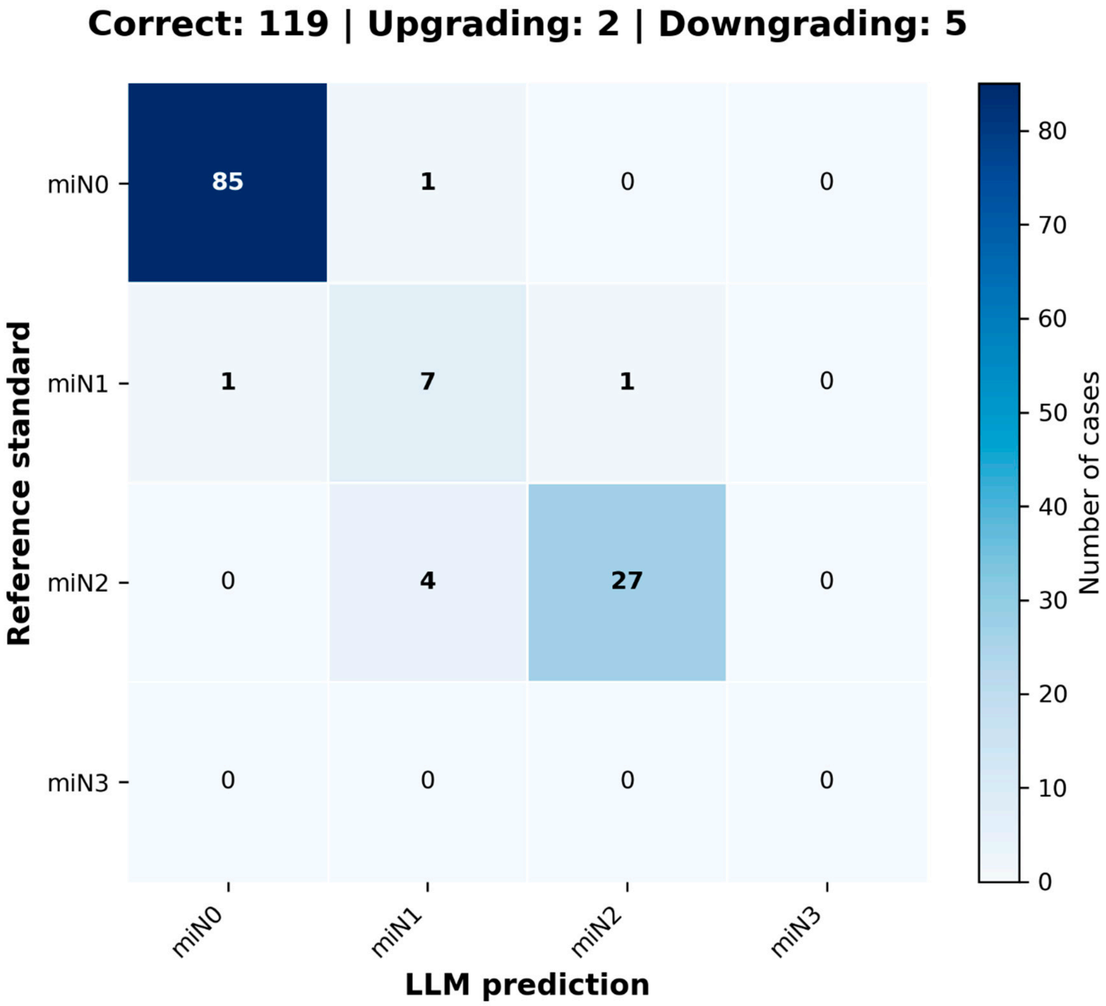

Figure S2. Confusion Matrix of GPT-5.4 for PROMISE Score V2 Classification (miN).

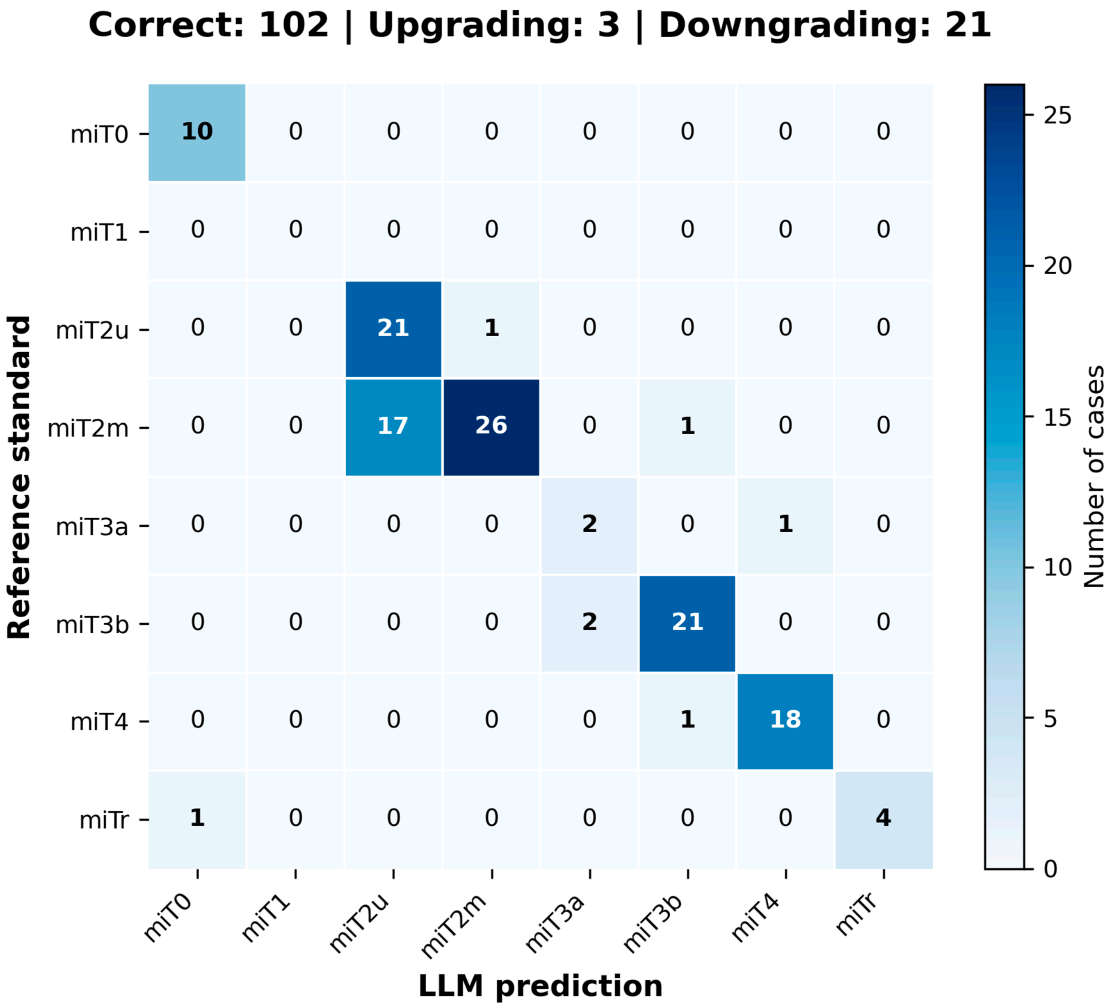

Figure S3. Confusion Matrix of GPT-5.4 for PROMISE Score V2 Classification (miT).

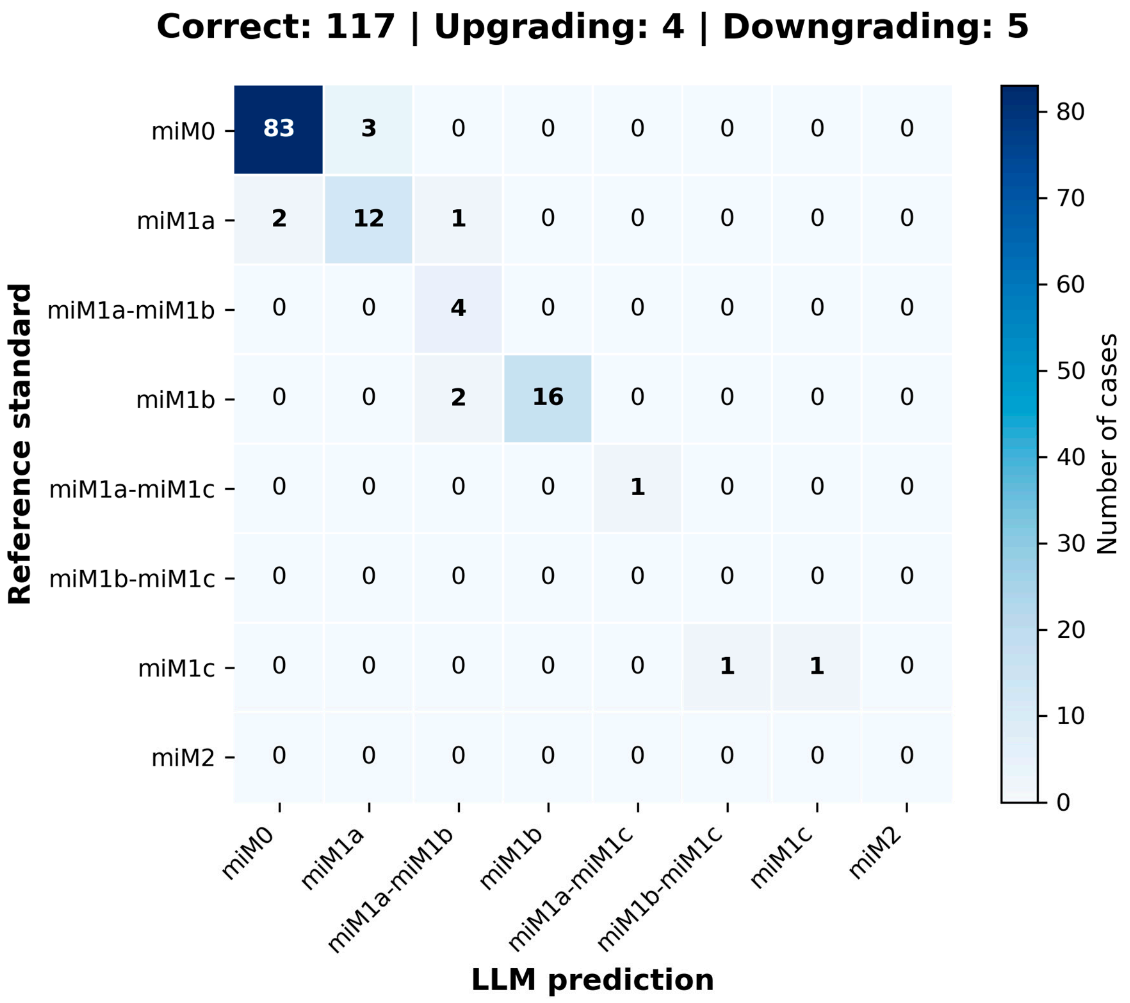

Reference standard \ LLM prediction	miM0	miM1a	miM1a-miM1b	miM1b	miM1a-miM1c	miM1b-miM1c	miM1c	miM2
miM0	83	3	0	0	0	0	0	0
miM1a	2	12	1	0	0	0	0	0
miM1a-miM1b	0	0	4	0	0	0	0	0
miM1b	0	0	2	16	0	0	0	0
miM1a-miM1c	0	0	0	0	1	0	0	0
miM1b-miM1c	0	0	0	0	0	0	0	0
miM1c	0	0	0	0	0	1	1	0
miM2	0	0	0	0	0	0	0	0

Figure S4. Confusion Matrix of Claude Sonnet 4.6 for PROMISE Score V2 Classification (miM).

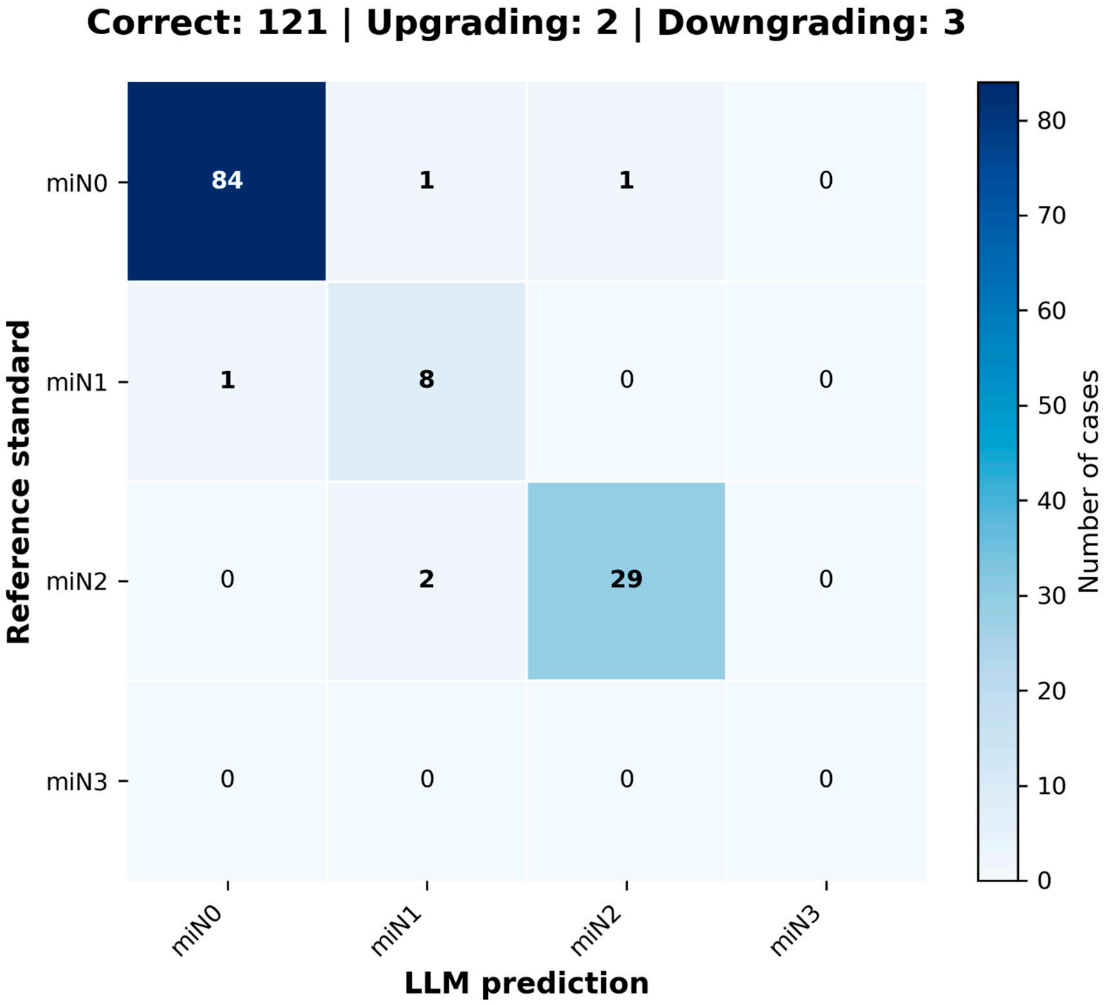

Figure S5. Confusion Matrix of Claude Sonnet 4.6 for PROMISE Score V2 Classification (miN).

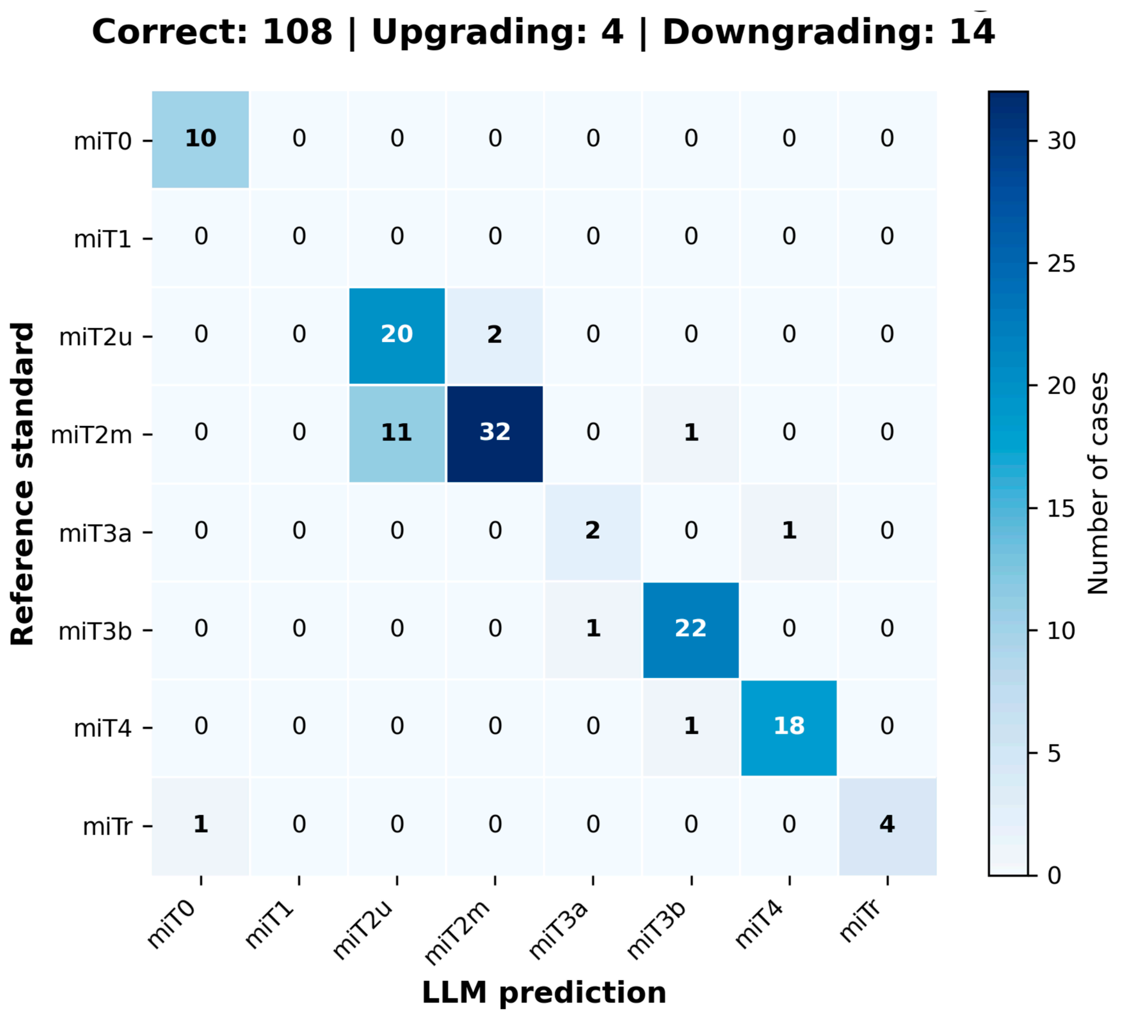

Figure S6. Confusion Matrix of Claude Sonnet 4.6 for PROMISE Score V2 Classification (miT).

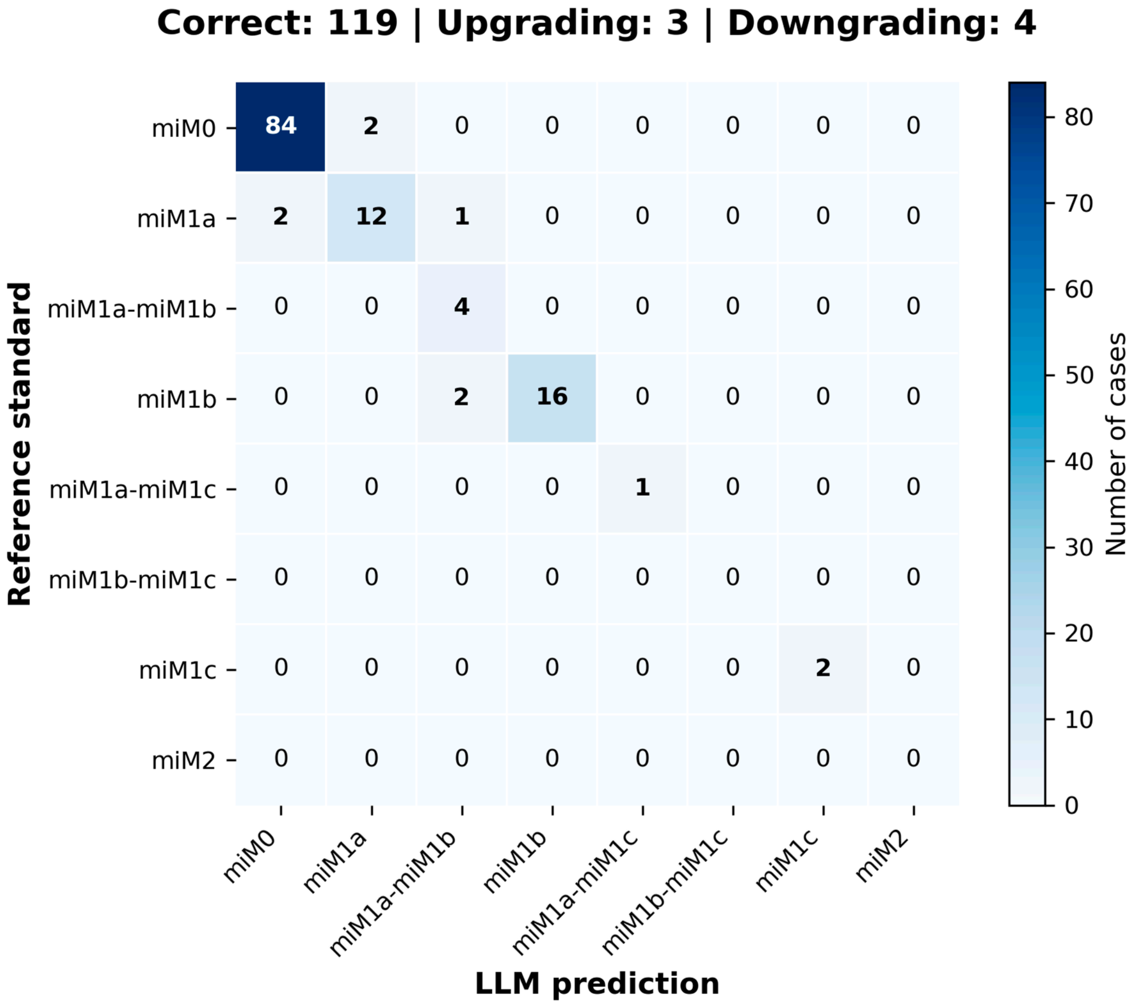

Figure S7. Confusion Matrix of DeepSeek V3.2 for PROMISE Score V2 Classification (miM).

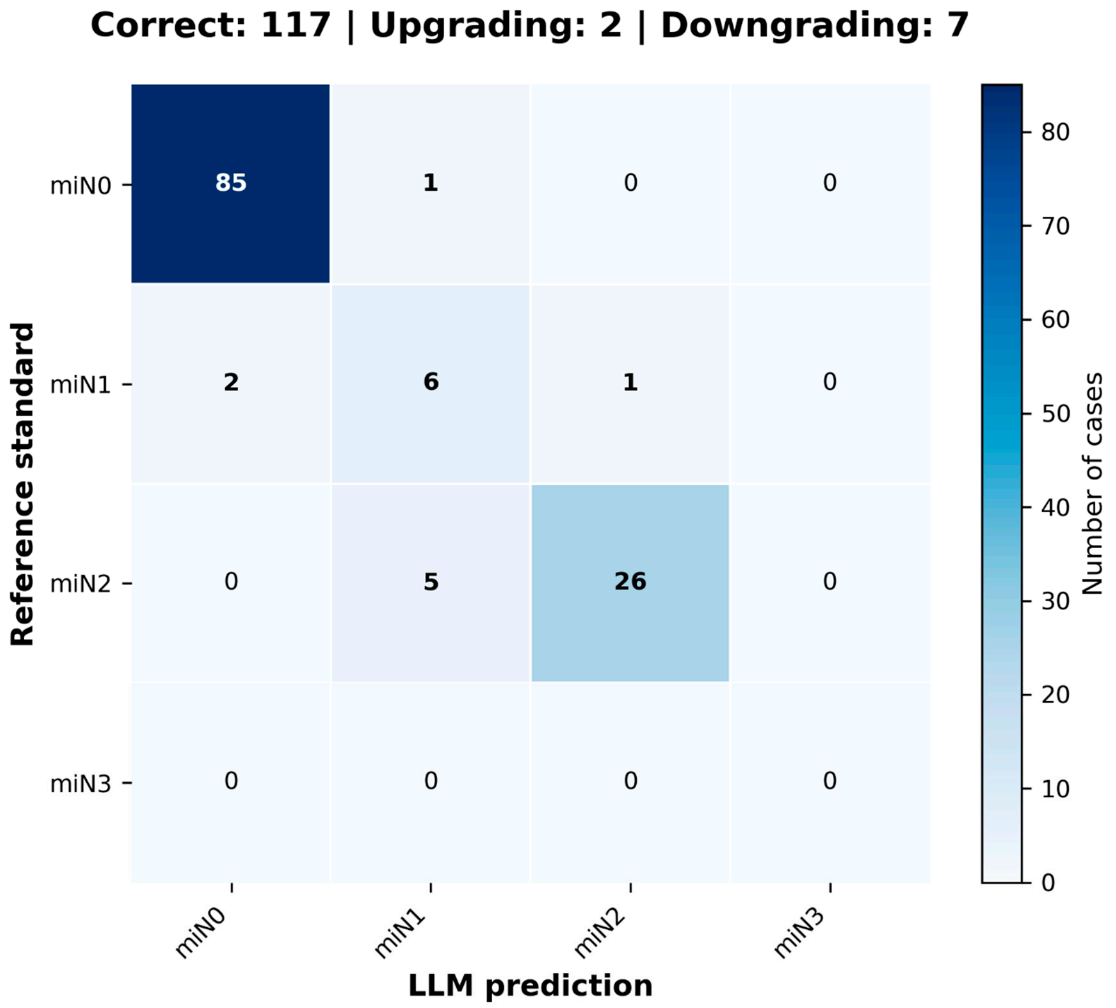

Figure S8. Confusion Matrix of DeepSeek V3.2 for PROMISE Score V2 Classification (miN).

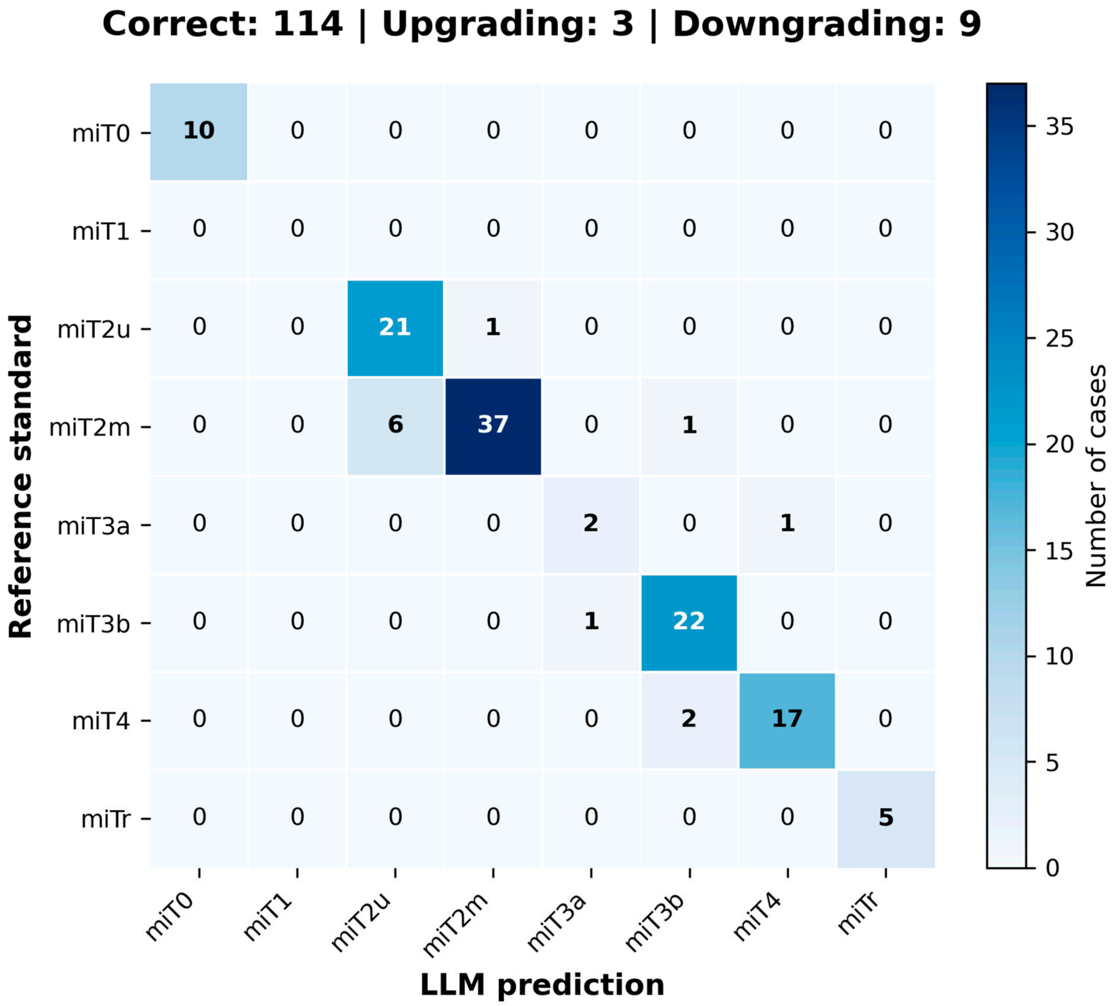

Figure S9. Confusion Matrix of DeepSeek V3.2 for PROMISE Score V2 Classification (miT).

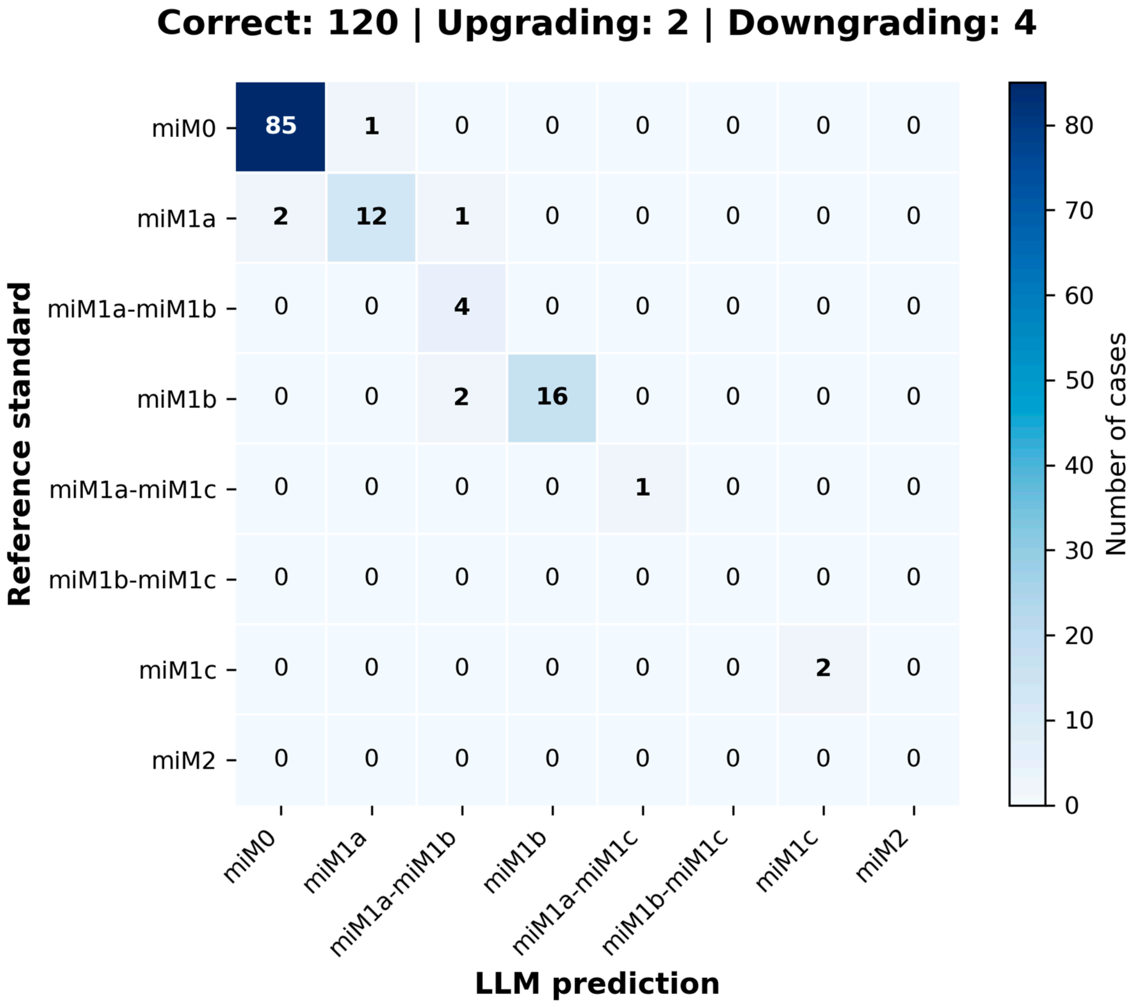

Figure S10. Confusion Matrix of Gemini 3 Flash for PROMISE Score V2 Classification (miM).

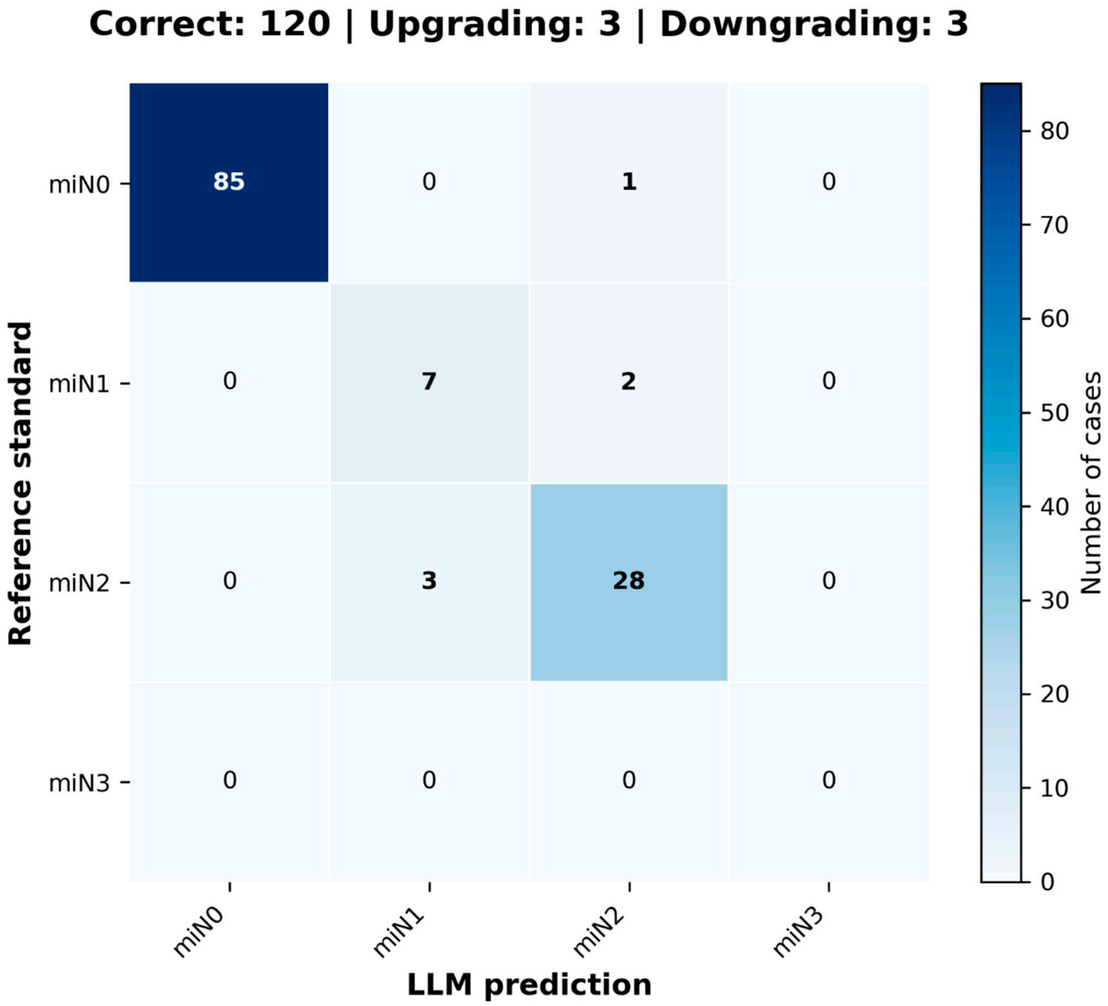

Figure S11. Confusion Matrix of Gemini 3 Flash for PROMISE Score V2 Classification (miN).

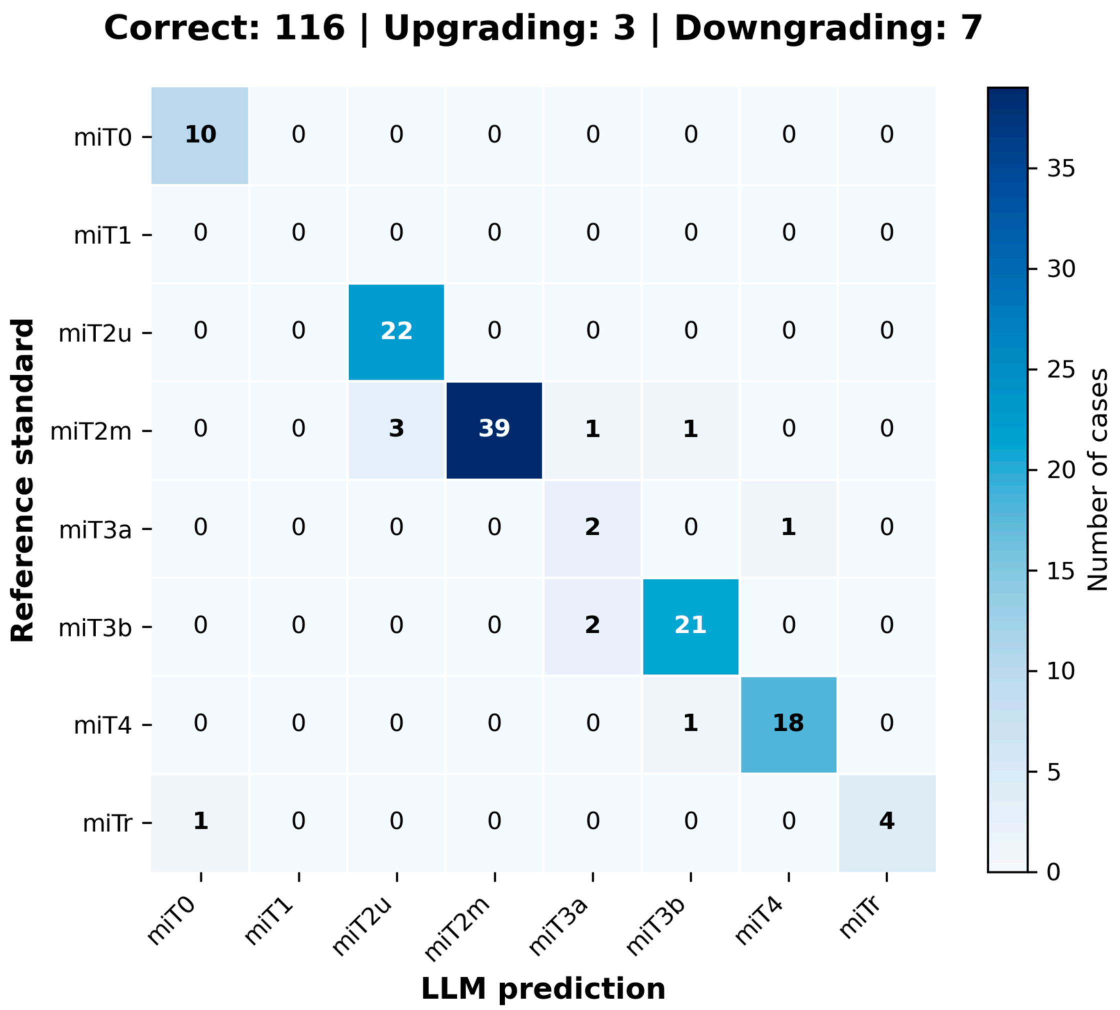

Figure S12. Confusion Matrix of Gemini 3 Flash for PROMISE Score V2 Classification (miT).

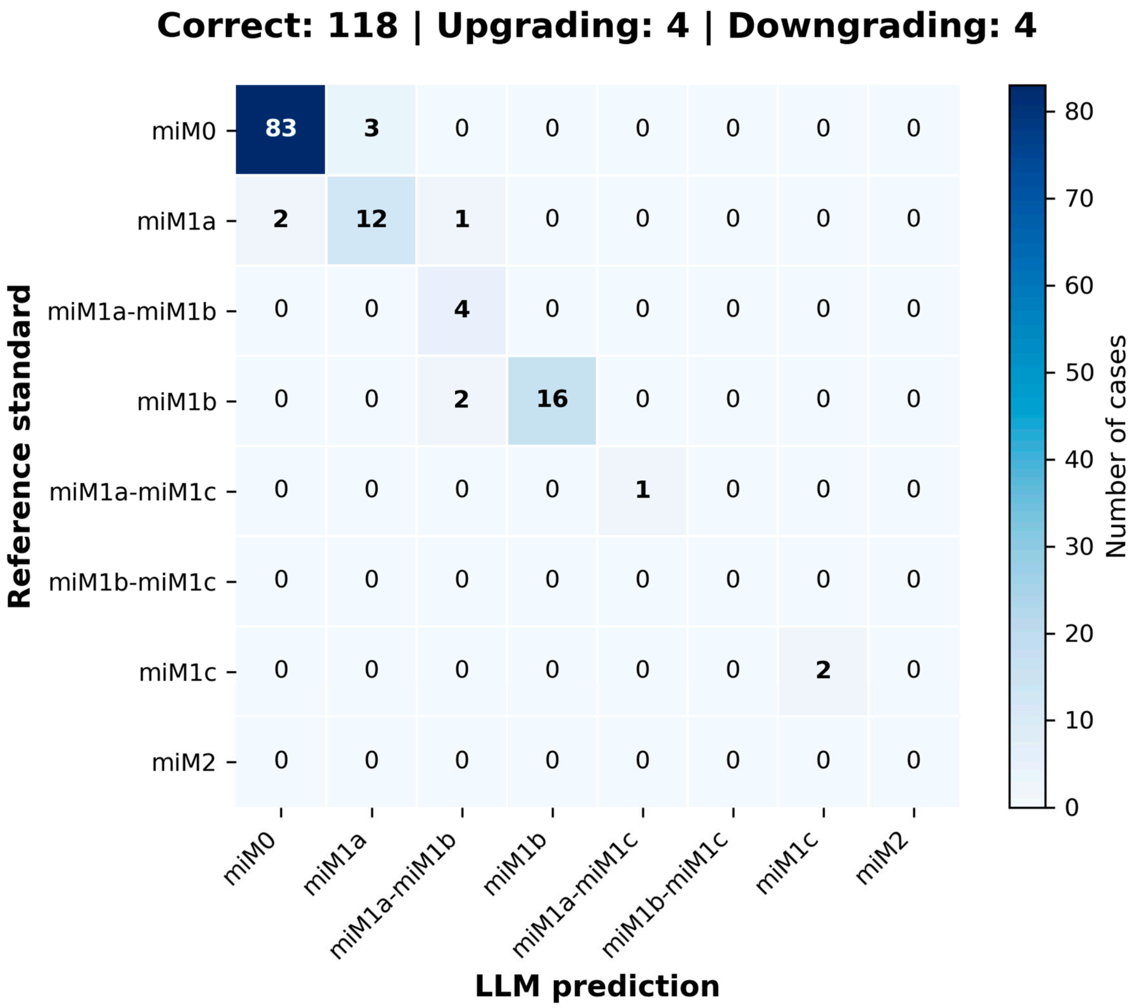

Figure S13. Confusion Matrix of Grok 4 for PROMISE Score V2 Classification (miM).

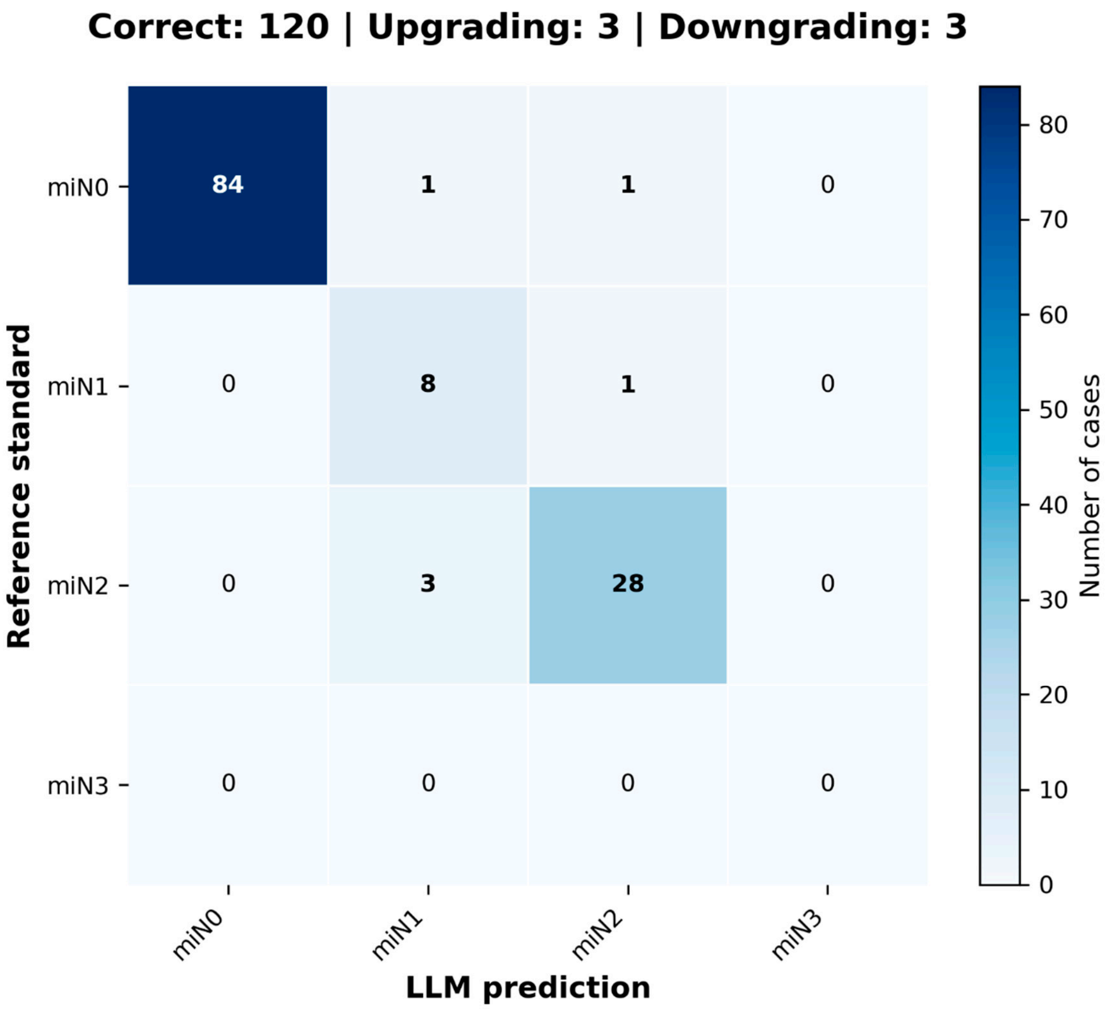

Figure S14. Confusion Matrix of Grok 4 for PROMISE Score V2 Classification (miN).

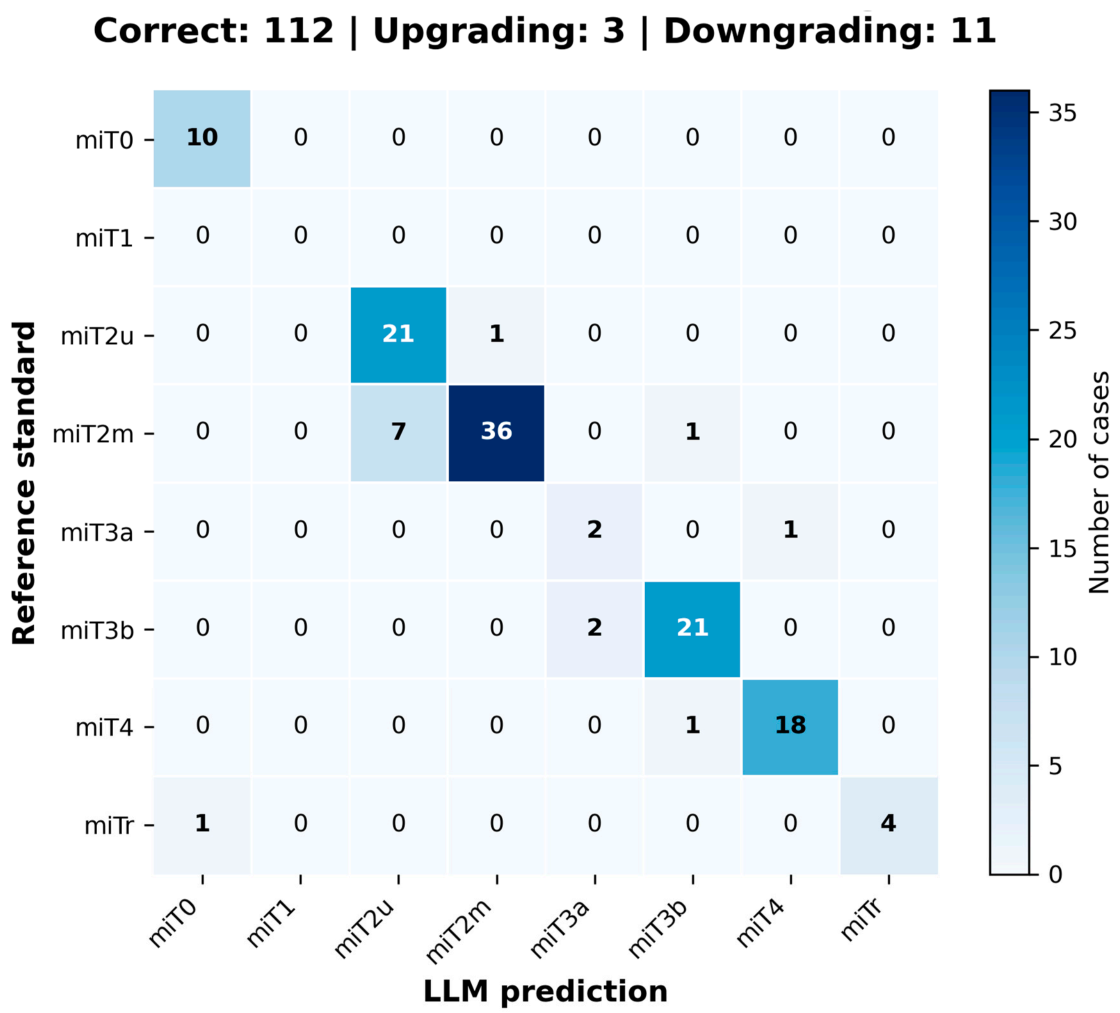

Figure S15. Confusion Matrix of Grok 4 for PROMISE Score V2 Classification (miT).

Short prompt (concise version):

Read the report and assign a PROMISE stage.
Return ONLY this JSON:

{"miT": "...", "miN": "...", "miM": "..."}
Use these values:

miT: ["miT0","miT2u","miT2m","miT3a","miT3b","miT4","miTr"]
miN: ["miN0","miN1","miN2"]
miM: ["miM0","miM1a","miM1b","miM1c","miM1a-miM1b","miM1a-miM1c","miM1b-miM1c","miM1a-miM1b-miM1c"]

miT:
- No tumor → miT0
- Tumor in prostate → miT2
- Extension outside prostate → miT3
- Invasion of other organs → miT4
- Recurrence after surgery → miTr

miN:
- No lymph nodes → miN0
- Some lymph nodes → miN1
- Many or both sides → miN2

miM:
- No metastases → miM0
- Lymph nodes outside pelvis → miM1a
- Bone metastases → miM1b
- Organ metastases → miM1c

OUTPUT: Return ONLY the JSON on a single line.
Examples:
{"miT":"miT3b","miN":"miN1","miM":"miM0"}
{"miT":"miT4","miN":"miN2","miM":"miM1a-miM1b-miM1c"}

Long prompt (detailed version):

You are a specialist in PSMA PET/CT reporting and PROMISE miTNM classification. Read the report and return the PROMISE miTNM stage as a single JSON object.Return ONLY this JSON, nothing else: {"miT": "...", "miN": "...", "miM": "..."}
Allowed values:
miT: ["miT0","miT2u","miT2m","miT3a","miT3b","miT4","miTr"]
miN: ["miN0","miN1","miN2"]
miM:
["miM0","miM1a","miM1b","miM1c",
"miM1a-miM1b","miM1a-miM1c","miM1b-miM1c","miM1a-miM1b-miM1c"]

FUNDAMENTAL RULE: BEURTEILUNG OVERRIDES BEFUND
The "Beurteilung" (impression) section is the ground truth.
Findings dismissed as "unspezifisch", "reaktiv", "a.e. degenerativ", "a.e. benign", "in erster Linie unspezifisch" → do NOT count as metastases.
Count only findings CONFIRMED or SUSPECTED (V.a., nicht auszuschließen) in Beurteilung.

miT CLASSIFICATION

PRIORITY ORDER: miT4 > miT3b > miT3a > miT2m > miT2u > miT0 (miTr = separate path)
── miT4 ── COMPLETE DECISION MATRIX
miT4 is assigned when ANY of the following apply:

TIER 1 — Explicit organ invasion (rectum, bladder, pelvic wall):
Confirmed: "Infiltration des Rektums", "Rektuminfiltration", "Blaseninfiltration", "Infiltration des Blasenbodens", "Infiltration der Beckenwand"
Cannot be excluded: "Rektuminfiltration nicht auszuschließen", "Blaseninfiltration nicht auszuschließen",
"Rektum- und Blaseninfiltration kann nicht ausgeschlossen werden"

TIER 2 — Mesorectal fascia with DIRECT infiltration wording:
"V.a. Infiltration der mesorektalen Faszie" → miT4
"Infiltration der mesorektalen Faszie" → miT4
"Verdacht auf Infiltration der mesorektalen Faszie" → miT4

TIER 3 — Seminal vesicle involvement COMBINED WITH fascia equivocal:
SVI (confirmed or suspected) AND "enge Lagebeziehung zur mesorektalen Faszie (hier Infiltration nicht auszuschließen)" → miT4
CRITICAL DISTINCTIONS:
"enge Lagebeziehung zur mesorektalen Faszie (hier Infiltration nicht auszuschließen)" WITHOUT SVI → miT3a
"V.a. SVI + enge Lagebeziehung zur mesorektalen Faszie" (no explicit "Infiltration nicht auszuschließen" for fascia) → miT3b
"methodenbedingt nicht sicher ausgeschlossen" → NOT miT4. Downgrade to miT3b/miT3a.

── miT3b ── [Samenbläschenloge ≠ Samenblase]
Seminal vesicle (Samenblase/Samenbläschen) itself involved — confirmed or suspected:
  "Infiltration der Samenblase/des Samenbläschens", "Samenbläscheninfiltration"
  "V.a. Infiltration der Samenblase", "Beteiligung der Samenbläschen"
  "Infiltration beider/bilateraler Samenbläschen"
"Samenbläschenloge" ≠ "Samenblase":
  "Samenbläschenloge" = anatomical region/bed → EPE (miT3a), NOT miT3b.
  Only "Samenblase" or "Samenbläschen" triggers miT3b.
If miT4 criteria also met → assign miT4, not miT3b.

── miT3a ──
EPE mentioned without SVI and without qualifying for miT4.
Phrases: "Kapselüberschreitung nicht ausgeschlossen", "V.a. Kapselüberschreitung", "dringender Verdacht auf Kapselüberschreitung", "extraprostatische Ausdehnung", "wandüberschreitendes Wachstum", "breitbasiger Kontakt zur Kapsel",
"enge Lagebeziehung zur mesorektalen Faszie" (WITHOUT "Infiltration nicht auszuschließen"),
"V.a. Infiltration der Samenbläschenloge" (Loge = EPE region)

── miT2m / miT2u ──
All lesions confined to prostate. No EPE, no SVI, no organ invasion in any form.
miT2u = 1 lesion; miT2m = 2+ lesions.

── miT0 ──
No intraprostatic lesion. In post-RP: explicit "Kein Hinweis auf Lokalrezidiv".

── miTr ──
Post-RP + local recurrence explicitly described in prostatectomy bed/anastomosis.
DECISION CHECKLIST (stop at first match):

1. Rectum/bladder/pelvic-wall invasion confirmed OR "nicht auszuschließen"? → miT4
2. "V.a. Infiltration der mesorektalen Faszie" (direct infiltration word)? → miT4
3. SVI confirmed/suspected AND "enge Lagebeziehung + Infiltration nicht auszuschließen"? → miT4
4. SVI (Samenblase/Samenbläschen, not Loge) confirmed or suspected? → miT3b
5. EPE mentioned in any form (including Lagebeziehung alone)? → miT3a
6. Multiple confined lesions? → miT2m
7. Single confined lesion? → miT2u
8. No lesion (native prostate)? → miT0
9. Post-RP + Lokalrezidiv described? → miTr
10. Post-RP + explicit "kein Lokalrezidiv"? → miT0

miN CLASSIFICATION
PROMISE miN — COMBINED LATERALITY + COUNT RULE:

miN0 = no confirmed pelvic LK metastases
miN1 = 1 or 2 pelvic LK metastases on ONE SIDE (unilateral), OR 1 confirmed pelvic LK
miN2 = pelvic LK metastases on BOTH SIDES (bilateral), OR ≥3 confirmed pelvic LK

KEY RULES:
- 1 LK ipsilateral → miN1
- 2 LK ipsilateral (same side) → miN1
- 2 LK bilateral (both sides confirmed) → miN2
- ≥3 LK on ANY side → miN2
- Bilateral = miN2 regardless of node count

Count rules:
- Count ONLY nodes confirmed or SUSPECTED in Beurteilung.
- Nodes dismissed as "unspezifisch" → NOT counted.
- CT-morphologisch suspekte LK with explicit "Verdacht auf PSMA-negative Lymphknotenmetastasen" in Beurteilung → DO count as metastasis.
- "ganz in erster Linie eine weitere LK-Metastase" = too weak, do NOT count.
- LK-Konglomerat = 1 station unless report gives explicit node count.
PELVIC = below aortic bifurcation: common iliac, external iliac, internal iliac, obturator, presacral, pararectal nodes within true pelvis.

INGUINAL LK = EXTRAPELVIC (→ miM1a, NOT miN)
Inguinal lymph nodes are classified as EXTRAPELVIC in PROMISE.

CRITICAL BOUNDARY — ILIACA COMMUNIS:
Common iliac (Iliaca communis / iliakal communis) lymph nodes are EXTRAPELVIC → classify as miM1a (CI), NOT as miN.
Only nodes strictly BELOW the aortic bifurcation count as intrapelvic (miN):
- internal iliac (iliakal intern / A. iliaca interna) → intrapelvic → miN
- external iliac (iliakal extern / A. iliaca externa) → intrapelvic → miN
- obturator (Obturator) → intrapelvic → miN
- presacral (präsakral) → intrapelvic → miN
- pararectal within true pelvis → intrapelvic → miN

EXTRAPELVIC = always miM1a (never miN):
- common iliac (iliakal communis / Iliaca communis / A. iliaca communis) → miM1a (CI)
- retroperitoneal / paraaortal above bifurcation → miM1a (RP)
- inguinal → miM1a (OE)
- mediastinal, hilar, supraclavicular, retroclavicular,retrocrural, cervical, axillary → miM1a

A patient can be BOTH miN1/miN2 AND miM1a simultaneously.
Do NOT promote miN because extrapelvic nodes exist — count them separately.
Do NOT ignore extrapelvic nodes because intrapelvic nodes are already present.

MIXED miN + miM1a SCENARIOS — STEP-BY-STEP GUIDE

When multiple lymph node regions are described, classify EACH node independently:

STEP 1 — For each reported LK: intrapelvic or extrapelvic?
Internal iliac, external iliac, obturator, presacral, pararectal → intrapelvic → miN
Common iliac, retroperitoneal (above bifurcation), inguinal → extrapelvic → miM1a

STEP 2 — Count ONLY intrapelvic nodes for miN:
0 confirmed/suspected intrapelvic nodes → miN0
1–2 nodes unilateral (one side) → miN1
bilateral (both sides) OR ≥3 nodes → miN2

STEP 3 — Check extrapelvic LK independently:
≥1 confirmed/suspected extrapelvic LK → set miM1a (regardless of miN result)
No extrapelvic LK → miM component stays miM0 (unless bone/visceral)

STEP 4 — Do NOT let extrapelvic nodes influence miN count or vice versa.
WORKED EXAMPLE:
Findings: retroperitoneal paraaortal left (L4) + left common iliac + left internal iliac
→ retroperitoneal paraaortal: extrapelvic → miM1a (RP) [does NOT count for miN]
→ common iliac left: extrapelvic → miM1a (CI) [does NOT count for miN]
→ internal iliac left: intrapelvic → miN counter = 1, unilateral
miN result: 1 intrapelvic node, unilateral → miN1
miM result: extrapelvic LK confirmed → miM1a
CORRECT: {"miN": "miN1", "miM": "miM1a"}
WRONG: {"miN": "miN2", "miM": "miM1a"} ← common iliac falsely counted as intrapelvic
WRONG: {"miN": "miN2", "miM": "miM0"} ← all nodes lumped into miN

miM CLASSIFICATION
IMPORTANT:
For miM evaluation, use only the base categories:
- miM1a
- miM1b
- miM1c
Ignore any subtype in parentheses for miM1b.

Examples:
- "miM1b (uni)" -> miM1b
- "miM1b (olig)" -> miM1b
- "miM1b (diss)" -> miM1b
- "miM1b (dmi)" -> miM1b
If multiple metastatic compartments are present simultaneously, return a hyphen-separated combination in this exact order:
miM1a-miM1b-miM1c

Allowed outputs for miM are ONLY:
- miM0
- miM1a
- miM1b
- miM1c
- miM1a-miM1b
- miM1a-miM1c
- miM1b-miM1c
- miM1a-miM1b-miM1c
Rules:
- Use "-" as separator

- Never combine miM0 with another value
- Keep canonical order: miM1a-miM1b-miM1c

Step-by-step:

1. If extrapelvic lymph node metastases are present -> add miM1a
2. If bone metastases are present -> add miM1b
3. If visceral metastases are present -> add miM1c
4. If none are present -> miM0

── miM0 ──
Explicit negative: "Kein Nachweis von Fernmetastasen", "Kein Nachweis von Lymphknoten- oder Fernmetastasierung", "kein Hinweis auf Fernmetastasen".
"Kein Nachweis ossärer oder viszeraler Metastasierung" alone does NOT mean miM0
if inguinal or other extrapelvic LK metastases are described.

── miM1a ── Extrapelvic LYMPH NODE metastases
EXTRAPELVIC LK LOCATIONS:
• retrokrural / retrocrural
• retroperitoneal / paraaortal above bifurcation
• mediastinal, hilär, supraklavikulär, infraklavikulär, zervikal, axillar
• inguinal

── miM1b ── BONE metastases
Osseous = rib, vertebra, ilium, pubic bone, femur, sacrum, sternum, skull, acetabulum.

── miM1c ── Visceral metastases
Visceral: liver, lung, adrenal, brain, peritoneum, mesentery.
OUTPUT: Return ONLY the JSON on a single line.
Examples:
{"miT":"miT3b","miN":"miN1","miM":"miM0"}
{"miT":"miT4","miN":"miN2","miM":"miM1a-miM1b-miM1c"}
